# Acute Respiratory Distress Syndrome and Fluid Management: Finding the Perfect Balance

**DOI:** 10.3390/jcm14062067

**Published:** 2025-03-18

**Authors:** Irene Sbaraini Zernini, Domenico Nocera, Rosanna D’Albo, Tommaso Tonetti

**Affiliations:** 1 Department of Medical and Surgical Sciences (DIMEC), Alma Mater Studiorum University of Bologna, 40126 Bologna, Italy; irene.sbaraini@studio.unibo.it (I.S.Z.); domenico.nocera@studio.unibo.it (D.N.); rosanna.dalbo@studio.unibo.it (R.D.); 2 Anesthesiology and General Intensive Care Unit, IRCCS Azienda Ospedaliero-Universitaria di Bologna, 40126 Bologna, Italy

**Keywords:** ARDS, fluid therapy, mechanical ventilation, heart–lung interaction, fluid responsiveness

## Abstract

ARDS is a challenging syndrome in which the hallmark is alveolar epithelium damage, with the consequent extravasation of fluids into the interstitium and alveolar space. Patients with severe ARDS almost always require mechanical ventilation and aggressive fluid resuscitation, at least in the initial phases. The increased intrathoracic pressure during positive pressure ventilation reduces cardiac output, worsening the circulatory status of these patients even more. In this pathological context, fluid therapies serve as a means to restore intravascular volume but can simultaneously play a detrimental role, increasing the amount of liquid in the lungs and worsening gas exchange and lung mechanics. Indeed, clinical research suggests that fluid overload leads to worsening outcomes, mostly in terms of gas exchange, days of mechanical ventilation, and ICU stay duration. For these reasons, this review aims to provide basic information about ARDS pathophysiology and heart–lung interactions, the understanding of which is essential to guide fluid therapy, together with the close monitoring of hemodynamics and fluid responsiveness.

## 1. Introduction

Acute respiratory distress syndrome (ARDS) stands as one of the most complex and demanding challenges for all clinicians in critical care. A very complex issue in its management is the correct use of fluids, an essential cornerstone of hemodynamic management and a potential cause of worsening lung functions. Maintaining oxygen delivery to vital organs while protecting the lungs and ensuring optimal gas exchange requires a delicate balance, a challenge where every intervention carries profound consequences, impacting patient outcomes. Starting from the pathophysiological background of ARDS, this literature review aims to summarize the available clinical evidence about fluid strategies, fluid responsiveness, and fluid therapy monitoring, trying to frame them into the interactions between lungs, heart, and vessels.

## 2. Pathophysiology

ARDS most often arises in bacterial or viral pneumonia (with fungal cases being less frequent). It can also develop due to inflammatory causes, such as pancreatitis; aspiration of gastric, oral, or esophageal contents, often complicated by secondary infection; and major trauma [1]. The main pathological characteristic of the syndrome is the increased permeability of the pulmonary endothelium, which allows fluids and proteins to leak into the interstitial space [2]. This causes interstitial edema, and the fluid then moves into the alveoli due to alveolar epithelium damage. Indeed, fluid reabsorption depends on the integrity of epithelial junctions and ion transport mechanisms, including Na^+^/K^+^-ATPase pumps, which are essential for fluid removal. This is further aggravated by the reduced lymphatic drainage of alveolar fluid [1]. Hydrostatic and osmotic forces predict the movement of fluids from the vascular compartment to the interstitial space, especially considering the damage to the extracellular matrix and endothelial glycocalyx [3]. As a result, the accumulation of fluid in the alveoli leads to impaired gas exchange and worsening of respiratory function. Additionally, the shift in fluid into the interstitial space leads to intravascular volume depletion, which can manifest as inadequate cardiac output with hemodynamic impact. Consequently, fluid strategies play a key role in a delicate balance between adequate support to circulation and avoidance of excessive pulmonary fluid accumulation.

### 2.1. Impact of Restrictive Versus Liberal Fluid Management on Lung and Distal Organ Damage

The appropriate approach to fluid management has been widely debated within the scientific community. Numerous studies demonstrate an association between fluid overload, subsequent hypervolemia, and glycocalyx damage [4,5,6]. Hypervolemia has been shown to negatively impact various aspects of patient outcomes, including cardiopulmonary complications, anastomotic insufficiency, prolonged hospital stays, increased duration of mechanical ventilation, and higher mortality rates [7,8]. One potential cause could be the release of atrial natriuretic peptide (ANP) from the cardiac atria due to mechanical wall stress during fluid overload. It has been shown that ANP can damage endothelial glycocalyx, which is essential for vascular health. In experimental models, it seems to increase vascular permeability, cause detectable degradation of the glycocalyx, and lead to significant tissue edema. This process contributes to the rapid shift in fluid from the blood vessels into the interstitial space [5]. Moreover, increased central venous and capillary hydrostatic pressures may reduce organ perfusion pressure and promote pulmonary interstitial edema. In experimental models of acute lung injury, a high fluid balance, characteristic of liberal fluid management, was observed to worsen perivascular edema and reduce lung gas volume. This occurs both due to direct damage to the alveolar–capillary barrier and to extrapulmonary causes, such as ascites, further compromising ventilation and perfusion [9,10]. Therefore, the main consequences of liberal fluid management may include pulmonary edema, reduced oxygen delivery, and damage to distal organs.

Organ damage can also be caused by insufficient fluid therapy. For example, an excessively restrictive approach can lead to renal hypoperfusion and further functional impairment. The restrictive fluid regimen in ARDS represents a therapeutic approach aimed at limiting excess fluid accumulation, with the goal of reducing the risk of pulmonary edema and improving oxygenation, preserving functional airspaces, and improving gas exchange efficiency. Also, limiting fluid overload helps prevent mechanical damage to the alveoli, known as ventilator-induced lung injury (VILI), which is particularly relevant in patients undergoing mechanical ventilation [11]. Despite these advantages, restrictive management can lead to significant pathophysiological consequences. Excessive reduction in fluid volume can decrease venous return and cardiac preload, lowering cardiac output and compromising organ perfusion. Renal hypoperfusion represents one of the most common complications, with the risk of acute kidney injury (AKI), as observed in both experimental and clinical studies [12,13]. Furthermore, severe fluid restriction can exacerbate hypotension in hemodynamically unstable patients, worsening circulatory instability. Reduced systemic perfusion can negatively affect microcirculation, promoting lactic acidosis and worsening the overall metabolic balance [14,15,16,17].

### 2.2. Heart–Lung Interactions During Spontaneous Breathing and Positive Pressure Ventilation

**Figure 1:**
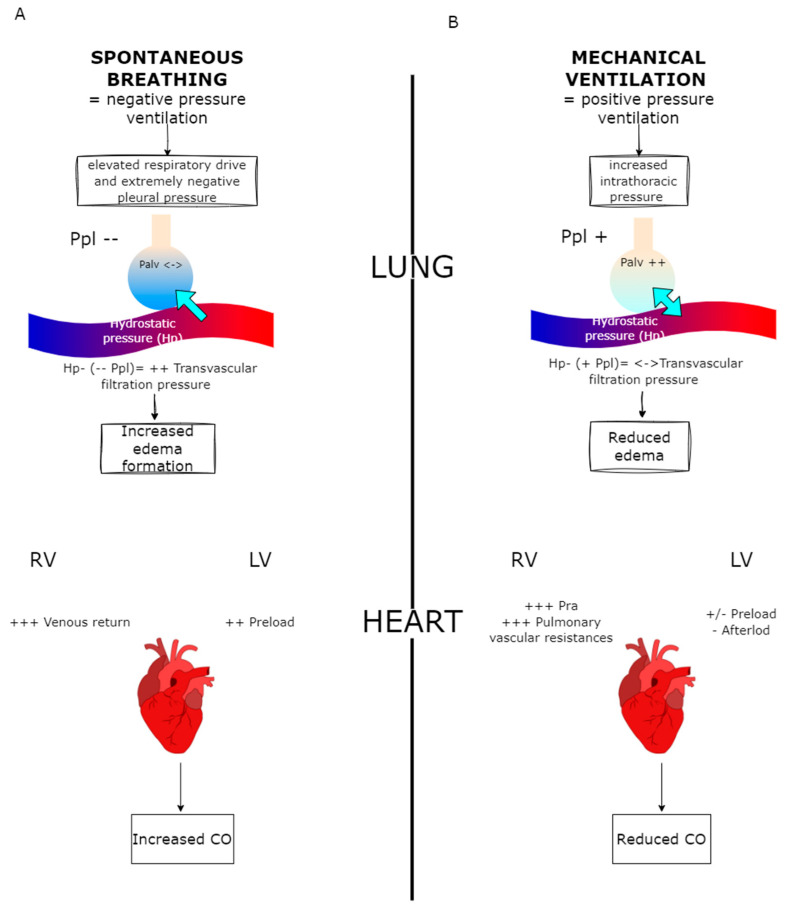
Description of heart–lung interaction. (**A**): During spontaneous breathing, especially with excessive inspiratory effort, negative Ppl (--) and increase transvascular filtration pressure, leading to edema formation (blue arrow). At the cardiac level, negative intrathoracic pressure enhances venous return (VR +++), overcoming the increased LV afterload and in turn increasing CO. (**B**): During mechanical ventilation, the increase in Ppl (+) and Palv (++), leads to a decrease in pulmonary edema formation (double-sided blue arrow). At the cardiac level, increased intrathoracic pressures reduce VR and increase RV afterload (+++ PVR). Although partially compensated by a decrease in LV afterload and by a potential increase in LV preload, this leads to an overall decrease in CO. Ppl: pleural pressure; Hp: hydrostatic capillary pressure; LV: left ventricle; RV: right ventricle; CO: cardiac output.

In addition to all the above mechanisms, fluid therapy should be inserted in the framework of heart–lung interactions. During ventilation, whether spontaneous or positive pressure, significant changes occur in pleural pressure, transpulmonary pressure, and lung volume, all of which influence hemodynamics and heart–lung interaction. This interaction is determined, among other things, by the thoracic location of these structures, where the heart and lungs are in close contiguity and connected through a network of vessels. Changes in intrathoracic pressure during respiration alter the pressure gradients that regulate blood flow to and from the heart [18]. Venous return to the right heart is influenced by the gradient between the mean systemic filling pressure (Pmsf) and right atrial pressure (Pra), whereas in the arterial compartment the pressure gradient is determined by the pressure generated by the left ventricle [19,20]. Although neither Pmsf nor arterial pressure is directly affected by isolated changes in intrathoracic pressure, cyclic fluctuations in the latter can alter pressure gradients, affecting atrial pressure and left ventricular transmural pressure. During spontaneous ventilation, pleural pressure is negative in both inspiration (more pronounced) and exhalation, promoting systemic venous return. This effect is reflected on the right atrium, affecting right ventricular preload and left ventricular afterload. In particular, right heart filling is inversely related to intrathoracic pressure, as venous return (and cardiac output) increases during inspiration due to the gradient between extrathoracic veins, which is determined by Pmsf and Pra. In addition, increased abdominal pressure during inspiration amplifies the pressure gradient (Pmsf–Pra) [21]. However, in pathological conditions such as ARDS, sepsis and asthma, significant changes in pleural pressure can be observed. In patients with ARDS, a marked increase in pleural pressure negativity related to elevated respiratory drive induced by inflammatory cytokines may promote pulmonary edema formation due to increased negative interstitial pressure and consequent increased transvascular filtration pressure (hydrostatic pressure-pleural pressure). In addition, intense inspiration can generate the pendelluft phenomenon, which accentuates lung stress and strain, exacerbating lung damage and further promoting edema formation [22,23]. During positive pressure ventilation, increased intrathoracic pressure significantly affects the circulation. The increase in airway, pleural, and transpulmonary pressure reflects on heart and vessels and reduces cardiac output for two reasons: venous return is decreased due to elevated Pra and right heart ejection is lowered by increased pulmonary vascular resistance [24,25]. This is only partially compensated in the left ventricle, where positive pressure ventilation reduces afterload by lowering transmural pressure (difference between ventricular and pleural pressures). In addition, left ventricular preload may increase due to elevated alveolar pressure, which promotes the movement of blood to the left heart. However, the net effect is a decrease in cardiac output, especially when high positive end-expiratory pressure (PEEP) and tidal volumes are used (Figure 1).

These effects are particularly evident in patients with cardiac failure. Indeed, during spontaneous ventilation, the increased transmural gradient increases left ventricular afterload, potentially compromising function in patients with left ventricular dysfunction. On the other hand, during positive pressure ventilation the previously said mechanisms promote ventricular emptying, an advantageous effect in patients with systolic dysfunction.

### 2.3. The Effects of PEEP

**Figure 2:**
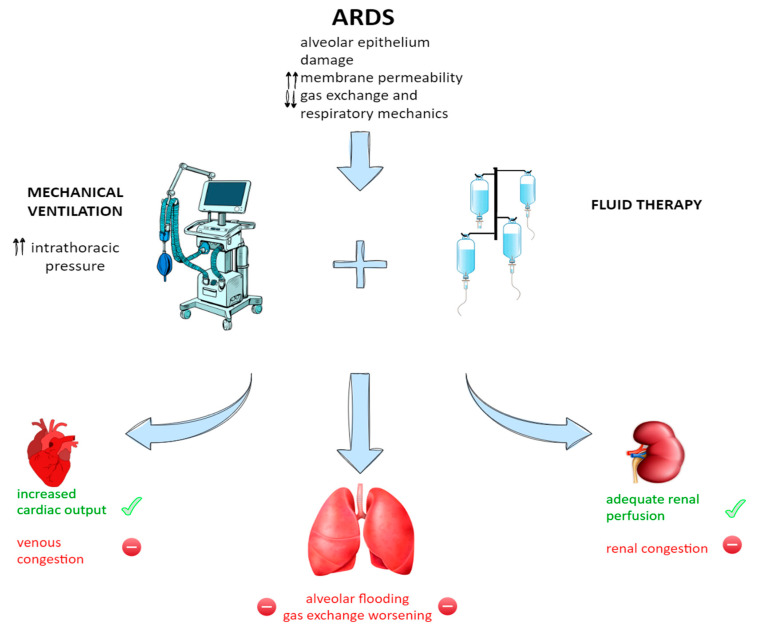
Pathophysiology of ARDS and consequences of its therapeutic management. The alveolar damage, the increased membrane permeability and the deteriorating gas exchange and respiratory mechanics happening during ARDS often require mechanical ventilation and aggressive fluid therapy. The interplay between them leads to consequences on the lungs, heart (and circulation), and distal organs such as the kidneys. ↑↑: increase; ↓↓ decrease.

For the explicated reasons, the necessity of maintaining adequate cardiac output often pushes clinicians to give large amounts of fluids in patients with ARDS, who are mechanically ventilated. Numerous experimental and clinical studies have highlighted complications arising from reduced effective circulating blood volume due to mechanical ventilation, especially when pleural pressure remains positive throughout the respiratory cycle, as occurs with the application of positive end-expiratory pressure (PEEP) [26]. This condition can lead to venous congestion and impaired organ perfusion, particularly affecting the kidneys, liver, and intestines. During intermittent positive pressure ventilation, a decrease in urinary output by 34%, glomerular filtration rate by 19%, renal blood flow by 32%, sodium excretion by 33%, and potassium excretion by 26% was observed. Notably, all of these alterations were fully reversed once PEEP was discontinued [27]. PEEP plays a central role both in its antidiuretic effects through renal vasoconstriction and in the stimulation of the renin–angiotensin–aldosterone system (RAAS), which may be mediated by mechanisms such as reduced renal perfusion pressure, altered sodium delivery to the macula densa, and increased renal neural activity. Additionally, decreased renal perfusion due to lower cardiac output and systemic resistance contributes to oliguria and fluid retention. The impact of PEEP on splanchnic blood flow is dose-dependent, as shown in animal studies. At PEEP levels below 10 cm H_2_O, reductions in blood flow are minimal, but they become more pronounced at levels of 15–20 cm H_2_O [28]. These changes can generally be reversed by supporting cardiac output with fluid resuscitation or the administration of inotropic and vasopressor agents. Although splanchnic blood flow decreases, oxygen consumption in the region is maintained, for an adaptive increase in oxygen extraction [29]. However, experimental results are not unequivocal. In fact, in an ARDS murine model, higher levels of PEEP associated with liberal fluid strategies worsened lung injury [30]. Moreover, an abrupt decrease in PEEP level was associated with an increase in alveolar damage markers, even worsened if combined with liberal fluid strategy [31] (Figure 2).

## 3. Discussion

### 3.1. Liberal vs. Restrictive Strategies in Clinical Studies

Given the complexity of heart–lung interactions and the added challenge of a demanding syndrome such as ARDS, several studies had the purpose of investigating the impact of different fluid strategies in acute lung injury. One of the first clinical studies suggesting the benefit of a “conservative” strategy took place in the late ‘80s [32]. It showed improved survival in patients who had a lower cumulative fluid balance. Shortly after, a retrospective analysis compared patients with ARDS with and without a 25% reduction in wedge pressure [33]. The group in whom there was this reduction showed lower mortality and lower ICU length of stay, although the latter is not statistically significant. These findings were confirmed by Mitchell et al., who investigated patients requiring PA catheterization, managing them based either on extra-vascular lung water (EVLW) or wedge pressure values. In the group in which the management was guided by EVLW, the fluid balance was significantly lower and the ventilation and ICU days fewer. Of note, the studied population was affected by pulmonary edema, regardless of the cause [34]. Then, during the first decade of the 2000s, the concept of protective ventilation and lower tidal volumes emerged. In these years, a prospective study found worse outcomes in patients with ARDS treated with higher tidal volumes and positive fluid balance, thus addressing for the first time the combined effects of fluid overload and deleterious mechanical ventilation as risk factors for adverse outcomes [35]. However, despite all this clinical evidence, until the FACT trial in 2006 there were no available RCT comparing different fluid strategies in patients with ARDS. The FACT is the largest study comparing conservative vs. liberal fluid management, although no targets for fluid balance have been individuated for the study [36]. Indeed, the unsolved problem in fluid therapy is the definition of what is conservative, what is restrictive and what is liberal. However, despite no significant difference in 60-day mortality, the conservative group had improved lung function and fewer mechanical ventilation and ICU days. The reduction in ventilator-free days was also in a subgroup of surgical patients, as shown by a post hoc analysis indicating that a conservative strategy is of benefit in ARDS irrespective of its primary cause [37]. Secondary outcomes of the FACT trial included renal failure and the need for dialysis. There were no differences between the two groups, suggesting no greater risk of extrapulmonary organ failure even when using restrictive fluid management. Two later post hoc analyses of this trial explored the incidence of AKI in the same population. Interestingly, one of them showed greater AKI incidence in patients managed with liberal fluid strategies and higher mortality [38]. In the other one, the use of diuretics after the diagnosis of AKI was associated with higher survival [39]. Differently from the renal side, an analysis on FACT trial survivors performed by telephone-based tests identified fluid restriction together with lower recorded PaO_2_ during the trial as possible risk factors for long-term neurocognitive impairment [40]. This could show a possible connection between a conservative fluid strategy and some later effect on neurocognitive abilities. Finally, the only sub-analysis of FACT patients showing a significant difference in mortality depending on fluid administration is a latent class analysis, identifying two ARDS subphenotypes with different responses to fluid management [41]. The hyperinflammatory phenotype (type 2) had reduced mortality when receiving restrictive fluids, while the hypoinflammatory (type 1) one showed the opposite, with reduced mortality with liberal fluid administration. The exact physiological reason is unknown, but perhaps the higher levels of inflammatory cytokines in phenotype 2 are responsible for augmented capillary permeability in these patients, with consequent enhanced alveolar flooding in case of fluid overload. This finding suggests a different response to fluid therapy based on different pathophysiological patterns. Later on, other studies confirmed the FACT trial results, highlighting the importance of avoiding fluid overload early from the beginning of the ARDS [42,43]. It has to be said, however, that patients with observed cumulative positive fluid balance are often the ones requiring initial aggressive use of fluid resuscitation and vasopressors [43]. Nonetheless, de-resuscitation of these patients after the critical initial phase could improve outcomes [44]. A meta-analysis including 49 studies on pediatric and adult patients affected by ARDS, Systemic inflammatory response syndrome (SIRS) and septic shock evaluating different fluid strategies, confirmed reduced mechanical ventilation days and ICU stays when using restrictive fluid therapies or deresuscitation, while results on mortality remain unclear [45]. Also, strategies aiming to achieve negative fluid balances with diuretic administration in addition to albumin infusion demonstrated to ameliorate oxygenation and hemodynamics variables, as compared with crystalloid infusion. This can be explained by an increase in the oncotic power of plasma, with consequently minor risks of alveolar flooding, and a parallel augmentation in the effective circulating volume [46,47]. Despite this potential pathophysiological rationale, current evidence does not demonstrate an improvement in mortality or ventilator-free days through the use of albumin combined with diuretics in mechanically ventilated patients. Based on this, the latest guidelines do not recommend this combination for the removal of extravascular fluids [48].

### 3.2. Practical Management and Fluid Therapy Monitoring

As already mentioned, in patients with ARDS, both hypovolemia and hypervolemia could be dangerous: the first reduces organ perfusion and the latter worsens lung function (gas exchange and mechanics impairment). In most cases, ARDS is not an isolated lung condition but is instead characterized also by circulatory failure. According to a four-hit model, shock develops through four different phases: resuscitation, optimization, stabilization (with focus on organ support), and evacuation (with a focus on organ recovery and the resolution of fluid overload) [49]. For this reason, the first initial step should detect the etiology of ARDS (either isolated or not). The second step should be the assessment of organ perfusion (mean arterial pressure, central venous pressure, capillary refill time, urinary output, lactates, central venous oxygen saturation, venous–arterial CO_2_ gap) to understand if an initial aggressive resuscitation is required. If this is the case, fluid therapy is essential to restore organ perfusion and early use of a vasopressor may be indicated. After achieving patient stabilization and mitigating the hemodynamic effects of mechanical ventilation, fluid management should be tailored to actual clinical requirements and informed by hemodynamic and tissue perfusion monitoring, in order to prevent the adverse consequences of fluid overload [49,50].

### 3.3. Fluid Responsiveness

Over recent years, many tests based on heart–lung interaction have been developed to evaluate fluid responsiveness (FR) [51]. Furthermore, transpulmonary thermodilution (TPTD) and critical care echocardiography (CCE) have become the most used bedside hemodynamic monitoring techniques, with the pulmonary artery catheter (PAC) reserved for specific conditions [50]. Each method has its limitations and recognizing these and integrating multiple hemodynamic monitoring approaches could support clinicians in tailoring fluid management to individual patient needs. Since it is well known that static markers of preload do not reliably determine FR, dynamic markers are recommended [52]. Stroke volume variation (SVV) and pulse pressure variation (PPV), the first dynamic parameters described, derived from arterial pressure waveform, reflect respiratory-induced variation in stroke volume, under constant arterial compliance. SVV and PPV greater than 15% are predictive of FR, but valid only under specific conditions: tidal volume (Vt) ≥ 8 mL/Kg, absence of spontaneous breathing or cardiac arrhythmias, and normal abdominal pressure [53]. The tidal volume challenge offers a way to overcome these limitations: by transiently increasing Vt from 6 mL/kg (protective ventilation) to 8 mL/kg, an absolute rise in PPV of ≥3.5% or SVV of ≥2.5% can serve as a reliable indicator of fluid responsiveness [35]. In the presence of spontaneous breathing, low lung compliance, low Vt or cardiac arrhythmias, fluid responsiveness can be evaluated in real-time through invasive TPTD monitoring. Several tests have been developed to detect FR mimicking a real fluid challenge: an increase in cardiac output (CO) ≥ 10% following a passive leg raising (PLR) [54], ≥8–10% after a Trendelenburg maneuver [55], ≥5% after an end-expiratory occlusion test (EEO) [56] or a mini fluid challenge (minimum of 100 mL over 60–120 s) [57]. Specifically, the EEO test, Vt challenge, and Trendelenburg maneuver might also be performed in the prone position [53]. In patients receiving mechanical ventilation with PEEP ≥ 10 cm H_2_O and no spontaneous breathing, reducing PEEP to 5 cm H_2_O may predict fluid responsiveness (FR) if an increase of 8.6% in cardiac index (CI) is observed [58] (Table 1). Two parameters derived by TPTD can also help the clinician in assessing the detrimental effects of fluid overload in patients with ARDS. The EVLW correlates with both the severity of lung edema and mortality, while the pulmonary vascular permeability index (PVPI) acts as a marker of capillary leak, distinguishing between inflammatory and hydrostatic edema [59]. CCE offers comprehensive hemodynamic and cardiac assessment, providing crucial insights especially in cases of right ventricular failure, where the aforementioned FR parameters may lead to potential clinical misjudgments. PPV is often associated with a high rate of false positives, particularly in patients with ARDS ventilated with elevated PEEP levels. In such cases, an increase in PPV after a fluid challenge or PLR may reflect right ventricular afterload dependence rather than fluid responsiveness [51]. A reduced peak systolic velocity of the tricuspid annulus, assessed using tissue Doppler imaging, can detect right ventricular dysfunction [60]. This situation is very common in patients with ARDS, with low lung compliance, increased transpulmonary pressure and acute cor pulmonale. Currently, the most specific parameter of FR is the dynamic variation in superior vena cava diameter (ΔSVC > 36%), although it requires the use of transesophageal echocardiography [61]. The inferior vena cava collapsibility index shares similar limitations with PPV, being prone to numerous artifacts and rendered unreliable in the presence of intra-abdominal hypertension [61]. Additionally, the end-expiratory diameter of the inferior vena cava has shown poor predictive value for FR in mechanically ventilated patients [53]. Another useful parameter for detecting FR is the aortic Doppler velocity–time integral (VTI), which reflects changes in left ventricular stroke volume. Variations in VTI following a passive leg raise (PLR) or combined end-expiratory and end-inspiratory occlusion tests can effectively assess FR in patients with ARDS [41,54]. While respiratory variation in VTI can also predict FR, it shares the same limitations as PPV [62]. Furthermore, the global assessment of venous congestion using the VExUS score has proven valuable as an indicator of fluid overload and the need for de-resuscitation [63].

A frequent reassessment and monitoring of FR allows for the final step in fluid therapy management of patients with ARDS: the evaluation of possible evacuation or de-escalation with the purpose of removing excessive fluids [49]. This can be achieved by the normal recovery from the disease, the use of diuretics or continuous renal replacement therapy (CRRT) [64].

## 4. Conclusions

In conclusion, ARDS is a dynamic syndrome characterized by evolving alterations in lung mechanics, vascular permeability, and systemic hemodynamics. In this setting, fluids play a paradoxical role, to be acknowledged in the frame of heart–lung interaction; they are vital to support circulation and prevent tissue hypoxia, yet every excess drop risks exacerbating alveolar flooding, impairing oxygenation, and worsening respiratory failure. The concept of “filling the tank” in ARDS comes with many challenges. Lung compliance, vascular recruitability, and the shifting interplay of systemic and pulmonary pressures redefine what the “tank” can hold. In ARDS, a one-size-fits-all approach is not only inadequate but also dangerous. It instead needs to be tailored depending on the patient’s clinical condition by measuring and continuously assessing lung mechanics and gas exchange, responsiveness to fluid strategy, and hemodynamic status. Thus, the daily question to ask patients with ARDS is no longer “how much fluid?” but rather “how much, when, and why?”.

## 5. Future Directions

There are essentially two relevant problems in the fluid management of ARDS: the heterogeneity of a syndrome like ARDS and the lack of studies focused on physiopathological mechanisms. Indeed, lung conditions (etiology, mechanics, and gas exchange), heart–lung interactions, and ventilation mode (spontaneous breathing, and assisted or controlled mechanical ventilation) differ among patients, so an epidemiological approach is not sufficient for providing adequate evidence for clinical practice. Therefore, it would be desirable for clinical research to focus on underlying mechanisms with a pathophysiological approach and not just on big data.

## Figures and Tables

**Table 1:** Main FR indexes, applications, and limitations in patients with ARDS.

Index	Main Diagnostic Threshold	Limitations	CO Monitoring	Spontaneous Breathing	Prone Position
Tests and Indices Derived from Heart–Lung Interaction					
PPV/SVV	≥15%	Vt ≥ 8 mL/Kg PBW False positives: cardiac arrhythmias, RV failure False negatives: IAH, low lung compliance, low Vt	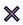	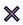	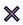
Vt challenge	↑ PPV ≥ 3.5% ↑ SVV ≥ 2.5%	Not reliable in cardiac arrhythmias, IAH	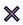	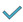	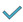
EEO test	↑ CO ≥ 5%	Need for 15 s EEO Not if breathing efforts or intense SB activity	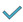	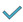	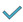
PEEP test	↑ CO ≥ 9%	PEEP ≥ 10 cm H_2_O Not reliable in: RV failure, IAH	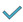	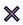	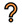
IVC distensibility index (dIVC)	dIVC ≥ 18%	False positives: SB activity, RV failure False negatives: IAH, low lung compliance, low Vt	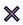	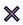	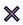
SVC collapsibility index (SVC-CI)	ΔSVC ≥ 36%	Need for TEE	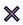	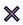	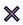
**Test Mimicking a Fluid Challenge**					
PLR	↑ CO ≥ 10% ↑ VTI ≥ 10% ↓ PPV/SVV ≥ 1–4 points ↑ etCO_2_ ≥ 2 mmHg	False negatives: IAH or venous compression stockings Not applicable if increased ICP	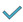	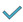	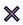
Trendelenburg maneuver	↑ CO ≥ 8–10%	Not reliable in: IAH Risk of gastric reflux Not applicable if increased ICP	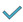	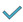	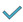
Mini–FC	↑ CO ≥ 5% ↑ VTI ≥ 10%	Fluid infusion (min. 100 mL)	** 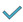 **	** 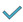 **	** 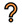 **

PPV: pulse pressure variation; SVV: stroke volume variation; Vt: tidal volume; EEO: end-expiratory occlusion; PLR: passive leg raising; FC: fluid challenge; IVC: inferior vena cava; SVC: superior vena cava; PBW: predicted body weight; RV: right ventricle; IAH: intra-abdominal hypertension; SB: spontaneous breathing; ICP: intra-cranial pressure; TEE: trans-esophageal echocardiography. ✓: yes; X: no; ?: not assessable.

## Data Availability

Not applicable.

## Abbreviations

The following abbreviations are used in this manuscript:

ARDS	Acute respiratory distress syndrome
ANP	Atrial natriuretic peptide
VILI	Ventilator-induced lung injury
AKI	Acute kidney injury
Pmsf	Mean systemic filling pressure
Pra	Right atrial pressure
PEEP	Positive end-expiratory pressure
RAAS	Renin–angiotensin–aldosterone system
EVLW	Extra-vascular lung water
SIRS	Systemic inflammatory response syndrome
TPTD	Transpulmonary thermodilution
CCE	Critical care echocardiography
PAC	Pulmonary artery catheter
FR	Fluid responsiveness
SVV	Stroke volume variation
PPV	Pulse pressure variation
CO	Cardiac output
PLR	Passive leg raising
EEO	End-expiratory occlusion test
PVPI	Pulmonary vascular permeability index
CI	Cardiac index
SVC	Superior vena cava
IVC	Inferior vena cava
